# Recent Advances in the Analysis of Phenolic Compounds in Unifloral Honeys

**DOI:** 10.3390/molecules21040451

**Published:** 2016-04-08

**Authors:** Marco Ciulu, Nadia Spano, Maria I. Pilo, Gavino Sanna

**Affiliations:** Dipartimento di Chimica e Farmacia, Università degli Studi di Sassari, via Vienna 2, 07100 Sassari, Italy; marcociulu@yahoo.it (M.C.); nspano@uniss.it (N.S.); mpilo@uniss.it (M.I.P.)

**Keywords:** unifloral honey, phenolic compounds, phenolic acids, flavonoids, honey classification, health properties, validation, chemometrics

## Abstract

Honey is one of the most renowned natural foods. Its composition is extremely variable, depending on its botanical and geographical origins, and the abundant presence of functional compounds has contributed to the increased worldwide interest is this foodstuff. In particular, great attention has been paid by the scientific community towards classes of compounds like phenolic compounds, due to their capability to act as markers of unifloral honey origin. In this contribution the most recent progress in the assessment of new analytical procedures aimed at the definition of the qualitative and quantitative profile of phenolic compounds of honey have been highlighted. A special emphasis has been placed on the innovative aspects concerning the extraction procedures, along with the most recent strategies proposed for the analysis of phenolic compounds. Moreover, the centrality of validation procedures has been claimed and extensively discussed in order to ensure the fitness-for-purpose of the proposed analytical methods. In addition, the exploitation of the phenolic profile as a tool for the classification of the botanical and geographical origin has been described, pointing out the usefulness of chemometrics in the interpretation of data sets originating from the analysis of polyphenols. Finally, recent results in concerning the evaluation of the antioxidant properties of unifloral honeys and the development of new analytical approaches aimed at measuring this parameter have been reviewed.

## 1. Introduction

Without any doubt honey is the most recognized and famous natural food produced by bees (*Apis mellifera*) from nectar and honeydew. Its historic, cultural and economic relevance make it the major beekeeping product [1]. It exhibits functional properties [2], and its significance in traditional medicine has been recognized in various cultures [3] and sacred texts like the Bible (“My son, eat honey, for it is good…”, The proverbs, 24:13) and Quran (honey is “... the healing for mankind”, 16:69). In principle, honey could be defined as an aqueous solution supersaturated in sugars (mainly fructose and glucose), but its chemical composition is much more complex and extremely variable, depending on a number of factors among which geographical and botanical origin are the most representative. Indeed, beyond glucose and fructose, it is possible to find many minor mono- and oligosaccharides [4,5], sometimes useful in order to gain information that helps identify the botanical origin of honey. Moreover honey is rich in hundreds of analytes other than sugars, usually present in a mass ratio between 10^−3^ and 10^−6^ (*w*/*w*), representing in principle a detailed “chemical fingerprint” that may be a very efficient tool for identifying both the botanical and geographical origin of honey as well as revealing adulterations or frauds [6]. In this context, it is worth remembering classes of compounds like nonaromatic organic acids [7,8,9,10], vitamins [11,12,13,14,15], free amino acids [16,17,18,19,20], inorganic elements [21] and—among others—phenolic compounds.

**Figure 1:**
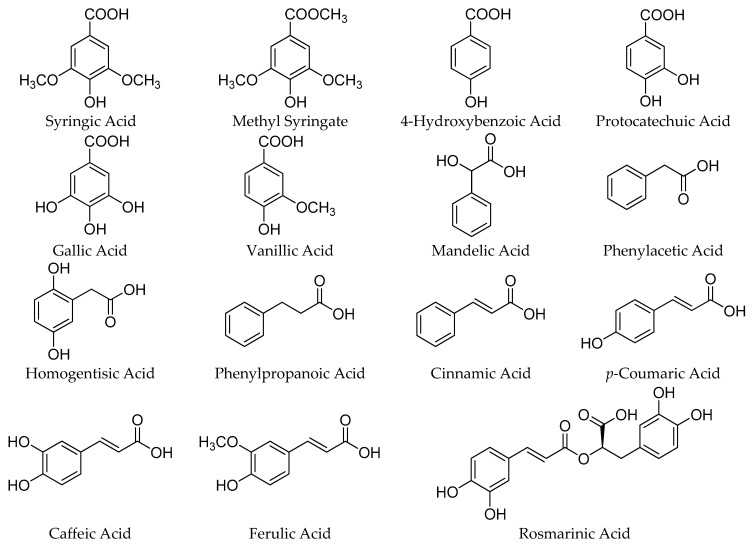
Phenolic acids and their derivatives in honey.

**Figure 2:**
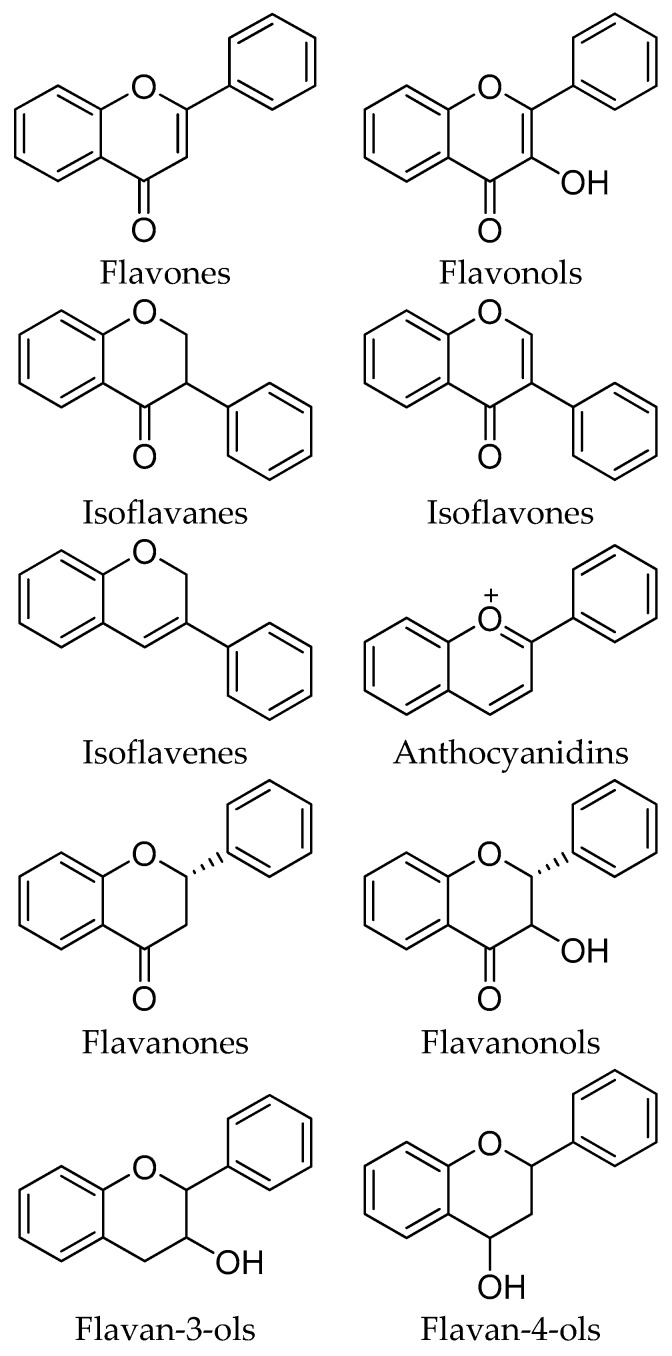
General structures of flavonoid subfamilies.

**Figure 3:**
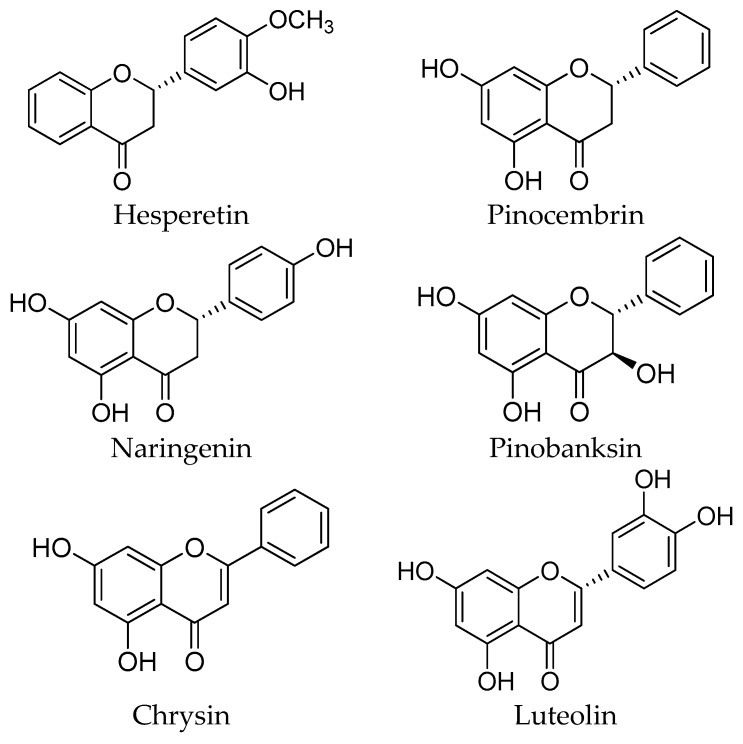

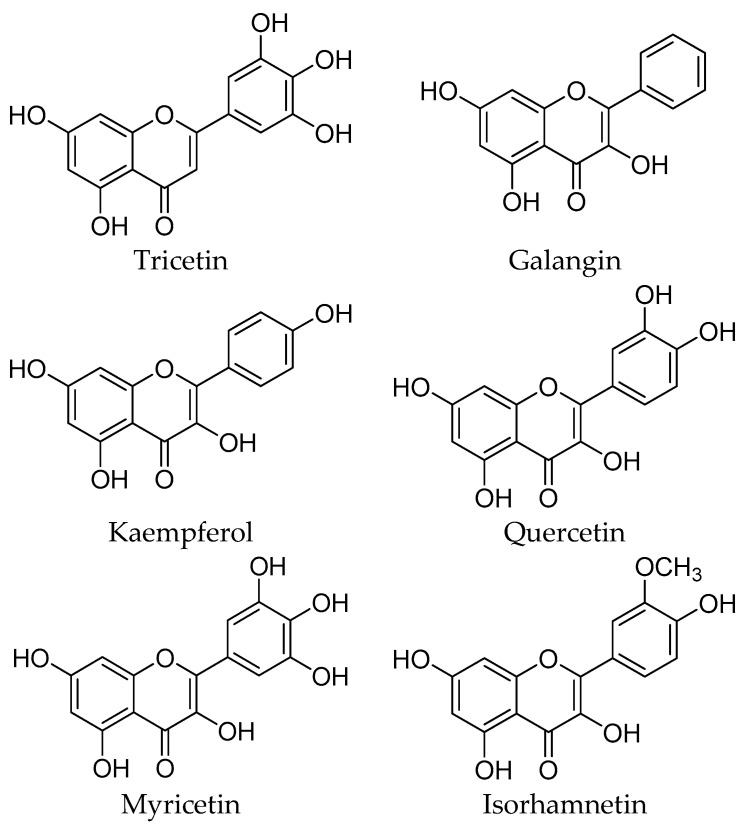
Most significant flavonoids in honey.

The most important sources of phenolic compounds in honey can be traced to the vegetal kingdom. These compounds are plant-derived secondary metabolites, biosynthesized mainly for protection against stress and oxidative damage and transferred via the nectar to the honey. The phenolic compounds of honey can be classified into two main families: phenolic acids with their related derivatives (Figure 1), and flavonoids (Figure 2 and Figure 3). While some of the most representative phenolic acids found in honey are shown in Figure 1, flavonoids, all characterized by the presence of an x-phenyl-1,4-benzopyrone backbone (where x = 2, 3), can be further classified in a number of subfamilies, reported in Figure 2, whereas a selection of the most important flavonoids identified in honey is available in Figure 3.

The qualitative and quantitative dissimilarities in the phenolic profile of honeys belonging to different floral sources are a direct consequence of the natural variability of these compounds in the plants from which they originate. This variability represents the scientific basis of the two main research themes regarding the study of honey phenolic fraction. The first approach is focused on the evaluation of the overall bioactive properties of honeys from different botanical (or—sometimes—geographical) origins, while the second one tries to attribute the floral and/or the geographical origin of honey on the basis of the presence and the abundance of one (or more) specific phenolic compounds, hence proposed as chemical marker(s) of origin. The outcomes of these studies are meaningful in both directions: honeys of different origins have shown a broad range of health-promoting properties like antibacterial, antioxidant and radical-scavenging activity [22,23,24,25,26,27]; on the other hand valuable results have been obtained in proposing a number of phenolic compounds as possible candidate markers of unifloral honeys [28,29,30,31,32,33,34,35,36,37,38]. In addition, first attempts of geographical attribution of honey according to differences in the phenolic profiles have been described in the literature [6,39].

The complexity of a food matrix like honey implies that the target analytes are usually present in low concentrations, and this demands the adoption of a multi-step analytical procedure able to provide a careful measurement of these quantities. In this context, the need to provide a proper validation protocol for the whole procedure of analysis in order to obtain reliable analytical data is nowadays felt much more than before [40].

Furthermore, the recent literature reports numerous attempts to provide a comprehensive view of the health-promoting properties of unifloral honeys and the attribution of their floral/geographical origin. In order to do this, different chemometric approaches have been used to obtain (or to process) analytical data of the phenolic profile in honey samples.

At the best of our knowledge, no recently published review provides a specific and updated state of the art on the analysis of the phenolic compounds in unifloral honey, related to the evaluation of its health-promoting properties and to the classification of its origin. Hence, the primary aim of this contribution is to fill this gap. Since the results of the less recent studies have been already reported in previous reviews [3,6,21,40,41,42], this article is primarily aimed at summarizing the pertinent studies carried out during the last decade. Within the chosen topics and this timespan, special attention has been devoted to studies where the quality of data produced is demonstrated by an adequate validation of the analytical method, and to those where a chemometric approach was used to manage analytical data and maximize the amount of information obtained.

## 2. Analytical Methods for the Determination of the Phenolic Profile of Unifloral Honey

### 2.1. General Remarks

The comprehensive characterization of the phenolic compounds of unifloral honeys generally begins with a proper sampling phase, aimed to obtain a large enough number of samples to provide representative results for a certain botanical and/or geographical origin. Obviously, the reliability of the information obtained from the phenolic pattern is strictly related to the authenticity and freshness of samples.

Usually floral source can be ascertained by means of melissopalynological analysis [43], while freshness can be proved quantifying the concentration of 5-hydroxymethyl-2-furaldehyde (HMF) [44,45], virtually absent in fresh samples, but whose concentration tends to increase after thermal treatments, improper storage or too long storage time.

For this reason, after sampling, honey should be stored in the dark and at low temperatures (typically 4 °C or less) until analysis, in order to preserve its chemical composition. Just before starting with the analysis, the analytical sample is allowed to reach the room temperature, and then it is homogenized. If sugar crystals are visible in the sample, they have to be dissolved by gentle heating, performed at temperatures never exceeding 40 °C.

According to the most of the published analytical procedures, and with only rare exceptions [38,46,47,48], phenolic compounds in honey need to be purified by means of both an extraction and clean-up phases followed by the separation and the identification steps, usually performed by chromatographic [28,29,30,31,32,34,35,36,37,38,39,40,41,42] or electrophoretic [33,41] approaches. The choice of the instrumental technique and the selection of the operative parameters strongly depend on the analytical goals and the type of characterization (qualitative and/or quantitative).

### 2.2. Extraction and Clean-Up

This represents a key step in the definition of the qualitative and quantitative profile of phenolic compounds in unifloral honeys. The aim of this phase is to guarantee an increased concentration of the target analytes and the simultaneous removal of any potential interfering compounds, such as sugars and other polar substances. The extraction and clean-up should represent the best compromise to maximize the recoveries for analytes, even when they belong to different chemical classes (flavonoids, phenolic acids, *etc.*).

**Figure 4:**
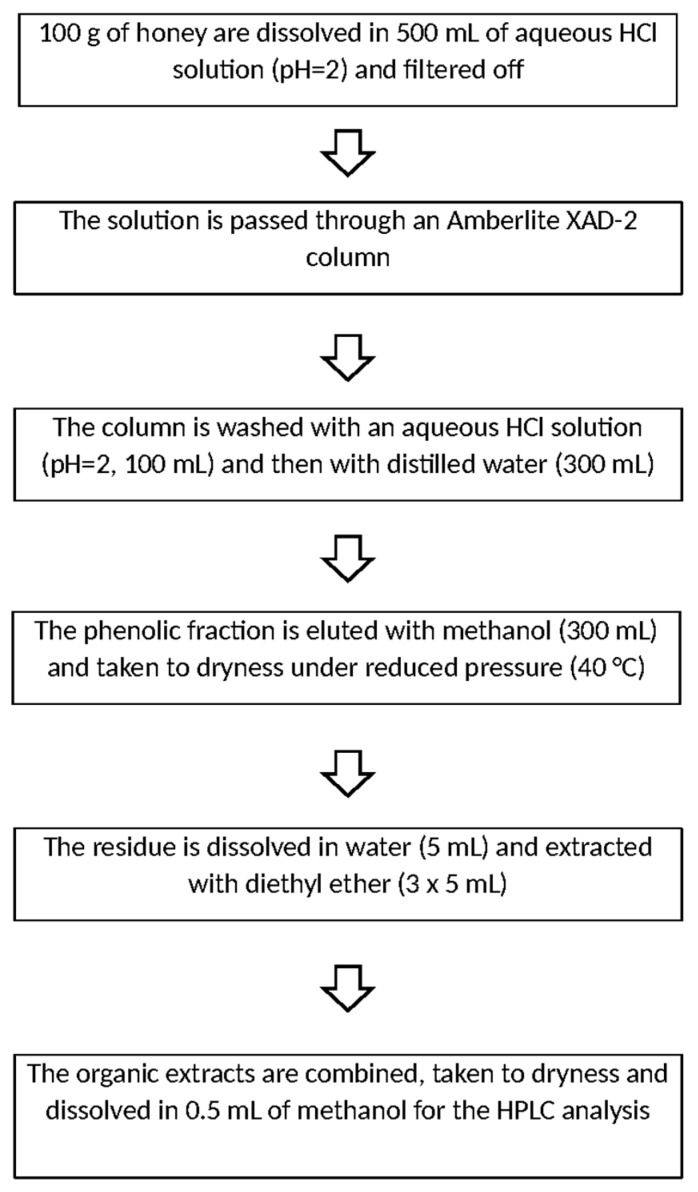
Extraction/clean-up of polyphenols from unifloral honey on Amberlite XAD-2, according [49].

In the last ten years, Amberlite XAD-2 resin has been one of the most popular adsorbent media for the extraction of phenolic compounds from honey. As described by Das and co-workers [49], the sample is dissolved in an aqueous solution of HCl (pH = 2), filtered and then passed through a column containing Amberlite XAD-2. Elution, accomplished first with aqueous HCl solution (pH = 2) and after with water, allows one to separate the phenolic fraction (retained on the column) from the polar interfering substances like sugars. Then, the analytes are eluted with methanol; the extracts are first evaporated to dryness at reduced pressure and then dissolved again in water. The clean-up phase can be performed by extraction with a proper organic solvent (diethyl ether [49] or ethyl acetate [50]). The organic extracts are finally evaporated and dissolved again in methanol for the HPLC analysis. The whole procedure is depicted in Figure 4.

**Figure 5:**
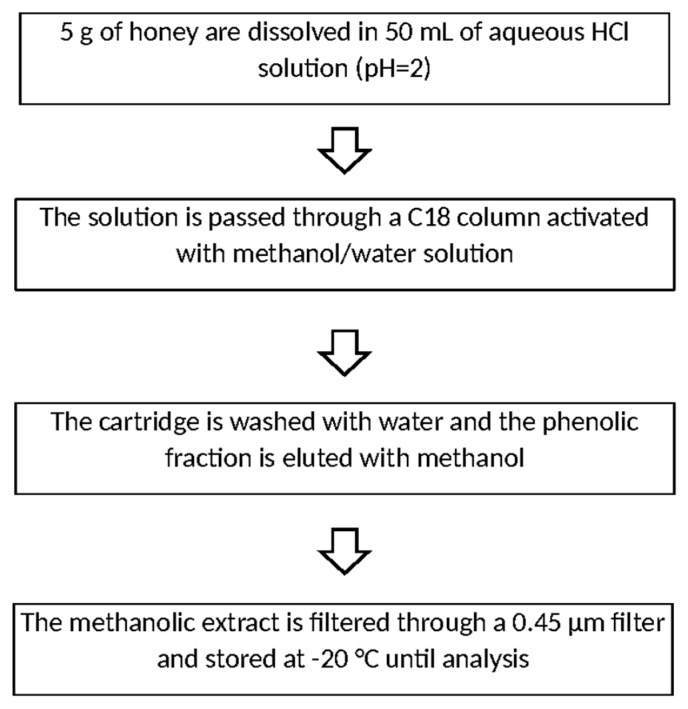
Extraction/clean-up of phenolic compounds from honey on C18 cartridge, according [51].

Some authors have chosen to simplify this step by adopting SPE methods, where phenolic compounds are retained by means of hydrophobic interactions with a solid sorbent. In this way it is possible to combine the extraction and clean-up phases, maximizing the recoveries and achieving considerable savings of time and solvents. As observed for the extraction with Amberlite, the sample is generally dissolved in acidified water. Prior its use, the SPE cartridge is washed and activated with a proper solvent mixture, depending on the nature of the sorbent phase (e.g., C18 [51,52] and Strata-X-SPE [53]). It is advisable that the sorbents strongly interact with a wide range of phenolic compounds. After the complete removal of polar interfering substances, the elution of analytes is usually performed with methanol. In Figure 5, the extraction/clean-up protocol using SPE cartridges, as described by Truchado and co-workers [51], is reported.

Michalkiewicz’s research group compared the performances of four sorbents (Bond Elut octadecyl C18, Oasis HLB, Strata-X and Amberlite XAD-2) for the isolation and preconcentration of six phenolic acids (gallic, *p*-HBA, *p*-coumaric, vanillic, caffeic and syringic acid) and three flavonols (rutin, quercetin and kaempferol) from honey samples. Oasis HLB sorbent phase, washed with 50 mL of acidified water (pH = 2) and eluted with methanol provided the best results [54]. Recently, Liu and coworkers proposed a new sorbent material, a nano-Al_2_O_3_ coated mesoporous silica (Al_2_O_3_/SiO_2_), to be used for the SPE of flavonoids. Its extraction properties were evaluated by using myricetin, quercetin, luteolin and kaempferol as the test analytes, and the extraction efficiency was apparently better than those of commercial C18 sorbents [55].

More consolidated procedures, like liquid-liquid extraction (LLE), have also been reported in recent studies [56,57] for the analysis of phenolic compounds in honey. In both cases, repeated extractions with ethyl acetate were performed on a solution obtained dissolving honey in pure water [56] or in a 2% NaCl aqueous solution [57]. While Tuberoso’s research group [56] performed, on the ethyl acetate extracts, a TLC clean-up aimed to isolate a specific phenolic compound (*i.e.*, the methyl syringate, proposed marker for the asphodel unifloral honey), the contribution of Kečkeš *et al.* [57] was focused on the definition of the phenolic profile of a number of Serbian unifloral honeys and, in this case, any additional clean-up phase was performed before the UHPLC-HESI-MS/MS analysis. More recently, Campone and co-workers reported an example of dispersive liquid–liquid microextraction (DLLME) of phenolic compounds from honey, obtaining recoveries normally higher than 70%. Subsequent analysis of these extracts were accomplished by means of a HPLC-UV method [58]. DLLME was also used in a similar way by Campillo *et al.* to determine flavonoid aglycones in honey using the HPLC-DAD-TOF-MS technique [59].

An appealing and recent improvement is represented by the use of multiwalled carbon nanotubes (MWCNTs) as sorbents for phenolic compounds [60,61]. MWCNTs are added to an acidified solution of honey, then the mixture is magnetically stirred in order to promote the adsorption of phenolic compounds onto the surface of the nanotubes. The sorbent material is first separated from the solution by vacuum filtration and then washed with water. Then, the treatment of the MWCNT with methanol causes the solubilisation of phenolic compounds. The methanolic solution is evaporated to dryness at 40 °C. The solid residue is dissolved with water and extracted three times with diethyl ether. The organic extract is evaporated to dryness, and the residue is finally dissolved in methanol for the HPLC analysis. The main advantages of this approach lie in the possibility to simultaneously extract a really wide number of phenolic species belonging to various classes (phenolic acids, flavonoids and the relevant derivatives) with high recoveries and reproducibility. Furthermore, the excellent regeneration properties of the MWCNTs let envisage their use for further extraction cycles of phenolic compounds in honey.

The literature also reports a number of contributions where no conventional extraction or clean-up procedure has been used. In these studies, the honey sample was analysed just after its solubilisation (in water [46,48], or in the HPLC mobile phase [47]) and the sonication for a few tens of minutes. This is the so-called “ultrasonic extraction” [62], that should not be confused with ultrasound assisted liquid–liquid extraction, extensively described in a review by Luque De Castro and Priego-Capote [63]). In this way Liang and coworkers [46] measured the amounts of four phenolic compounds (*i.e.*, caffeic acid, *p*-coumaric acid, ferulic acid, and hesperetin) in Chinese citrus honey by HPLC-ECD. The low number of analytes measured, the selectivity and sensitivity of the detector and the absence of a complex pre-treatment of the matrix allowed the authors to achieve almost quantitative recoveries for the compounds considered. An ultrasonic extraction technique was also used by Zhang *et al.* [47] to assess a very interesting multivariate calibration technique (second-order calibration method based on a trilinear decomposition algorithm) in the development of a HPLC-DAD method aimed at the quantification of nine polyphenols in five unifloral honey samples. This calibration technique involves the mathematical decomposition of the overlapped chromatographic profile into the pure profiles of each chemical species even in the presence of unknown interferences or uncompleted chromatographic resolution of peaks, overcoming also the problem of the baseline drift. According to the authors, the procedure is characterized by rapidity (the whole chromatographic run is completed in less than 8 min), linearity and good recoveries (within the range 90%–110% for all analytes). In a similar way, in a paper concerning the HPLC-UV determination of thirteen phenolic compounds in Italian honeys [48], samples were simply dissolved with distilled water and placed in an ultrasound bath at 25 °C for 10 min. Despite of the absence of any control system for the column temperature (not reported in the experimental section, and confirmed by the inconsistency of the retention times displayed in the published chromatograms), and notwithstanding the unsatisfactory chromatographic resolution, the authors reported outstanding recovery performances (ranging—for all analytes—from 98.50% for *p*-coumaric acid to 100.80% for quercetin) and precision (RSD values never exceeding 3%).

In a recent study published by Biesaga and Pyrzynska [62], aimed at checking the stability of phenolic compounds in ultrasonic- or microwave-assisted extraction, the authors demonstrated that the ultrasound-assisted extraction normally provides better extraction yields than conventional extraction performed by shaking immiscible solvents. However, the ultrasonic extraction of aglycones of flavonols (like quercetin) shows, under these conditions, very low yields (recovery values always less than 2% for different honey samples), due to their instability. 

Despite the growing interest inthe application of advanced liquid extraction techniques aimed to obtain phenolic extracts, only one example of accelerated solvent extraction (ASE) has been found in the literature during the last ten years [64]. The extraction is performed dissolving the sample in acidified water (pH = 2, HCl), at 25 °C by means of four different static cycles. Polyphenols are eluted with methanol, the solution is evaporated until dryness, and the residue is resuspended in distilled water and extracted three times with diethyl ether. Extracts are again dried and dissolved in a methanol/water solution before the HPLC analysis.

### 2.3. Analysis

Separation, identification and quantification are the next steps towards the definition of phenolic profile of unifloral honeys. The most used analytical technique is very often a HPLC approach, almost always in its reverse phase configuration (RP-HPLC). Selected features of recent HPLC methods used for the analysis of honey phenolic extracts have been summarized in Table 1.

The separation of the analytes is usually achieved by the use of C18 (ODS) columns, except for the Discovery HS PEG [59], a polyethylene glycol reversed phase column. In the last years, the application of ultra-high performance liquid chromatography (UHPLC) systems has proved advantageous in the analysis of the phenolic constituents of honey, improving the resolution, sensitivity and accuracy of the methods and reducing time of analysis [56,58,61,67]. The reason for the better analytical performances lies in the smaller size of the stationary phase particles (usually smaller than 2 µm) and the possibility to deliver the liquid phase at very high pressures. The mobile phase is generally composed of a gradient of two solvents: A) an aqueous acid solution of formic acid [47,50,51,53,57,58,59,60,61,67] or, alternatively, acetic acid [46,64], trifluoroacetic acid [65,66], phosphoric acid or phosphate buffers [48,49,52,56] and B) methanol or acetonitrile. 

The most used HPLC detection system for measuring the phenolic profile of unifloral honeys is still based on the measurement of the UV absorption, sometimes performed using diode-array devices (DAD) [47,50,51,56,60,61,65,67,68]. The choice of appropriate absorption wavelengths is fundamental to maximize the method sensitivity, especially when the target compounds do not belong to a specific class. Unfortunately, HPLC-UV identification of polyphenols is possible only by the comparison of retention times and by the peak spiking method, and these ways are practicable only when the analyte under quantification is effectively available as chromatographic standard. When the molecular detection is performed by a diode-array spectrophotometer, also the whole UV-Vis spectrum can contribute to the identification of peaks. The principal drawback of all spectrophotometric detectors is the inability to provide a direct structural information, and this strongly limits the characterization of compounds which were not previously identified. For this reason, in the last years many studies have been focused on the assessment of methods that involve different couplings between HPLC and mass spectrometry.

Among others, the most common instrumental combinations used are UHPLC-MS^n^ (1 ≤ *n* ≤ 3) [57,61], HPLC-MS^n^ (1 ≤ *n* ≤ 3) [56,69,70,71,72], HPLC-DAD-MS^n^ (1 ≤ *n* ≤ 3) [51,59,73] and UHPLC-DAD-MS^n^ (1 ≤ *n* ≤ 3) [67,74]. The growing interest in the exploitation of mass spectrometry and tandem-mass spectrometry as detection systems in HPLC led to the identification of new possible markers for honeys from specific botanical origins, like the methyl syringate for asphodel honey [56]. Moreover, the study of the fragmentation patterns allows performing the structural investigations on particular classes of compounds whose discrimination is made difficult by the strong similarities in their structures. In a recent study performed by Truchado and coworkers [71], the HPLC-ESI-MS^n^ characterization of *O*-glycosyl flavones of honeys produced by *Tetragonula carbonaria* bees consented to determine the nature of the inter-glycosidic linkage and to identify several flavonoid mono-, di- and triglycosides. Both ion trap and triple quadrupole mass spectrometers, along with the more recent hybrid instruments, have been used for the characterization of the phenolic fraction of honey. The ESI source, which is the most frequently installed in this kind of spectrometers, is generally set on the negative ion mode but also the positive mode can be helpful for the identification of some of the analytes [53,73].

Heated electrospray ionization probes (HESI) can be used to enhance desolvation during the ionization phase improving sensitivity. For example, this expedient was adopted by Kečkeš *et al.* [57] for the identification of polyphenols and other phytochemicals in Serbian unifloral honeys. In addition, Petrus and coworkers performed the determination of seven flavonoids in unifloral honeys by HPLC coupled with coulometric electrode array detection (CEAD), but confirmed the previously made attributions with HPLC-ESI-MS evidences [72].

Only some rare examples of analytical methods assessed by the use of techniques different from the traditional RP-HPLC are reported in the scientific literature. In this context, it is possible to consider the study by Jandric’s research group [83], performed by means of a multimethodological analytical approach (*i.e.*, an analysis of the elements, a stable isotope analysis, metabolomics findings, and NIR, FT-IR, and Raman spectroscopic fingerprinting) and chemometric instruments, the contribution of Sergiel *et al.* [84], aimed to explore the suitability of a right-angle geometry three-dimensional synchronous fluorescence spectroscopy for the differentiation and classification of unifloral honeys, and the recent results obtained by Lenhardt and coworkers [85], that coupled fluorescence measurements with parallel factor analysis and partial least squares discriminant analysis for the characterization and classification of honey of different botanical origin. 

### 2.4. Validation

Validation of an analytical method represents an essential component of the measures that any laboratory should implement to produce reliable analytical data. According to the 2nd Edition (2014) of *The Fitness for Purpose of Analytical Methods Eurachem Guide* [86], validation “…is basically the process of defining an analytical requirement, and confirming that the method under consideration has capabilities consistent with what the application requires.” According to ISO/IEC 17025 [87], a method should be validated whenever it is: (a) a non-standard method; (b) a laboratory-designed (or developed method); (c) a standard method used outside its intended scope; (d) an amplification and/or modification of standard method. Even if outside of the field of application of ISO/IEC 17025, some sectors (including food analysis) are anyway subject to validation requirements prescribed by international organizations like EC [88] or FAO [89]. 

These introductory definitions and considerations should provide sufficient support of the mandatory need, for any analytical chemist involved in food analysis, to provide—whenever necessary, and certainly when a new analytical method is proposed—at least the key parameters of a validation protocol, *i.e.*, limits of detection (LOD) and quantification (LOQ), the working range, the precision and the trueness. 

Although nowadays the attention for the presence of basic validation data in scientific publications is surely higher than in the last years, unfortunately more than 30% of the methods reported in Table 1 are completely unvalidated and this fact must raise some doubts on the reliability on any of the analytical data there reported.

As regards methods including some validation parameters, it is evident that precision and—above all—trueness play a key role for the correct interpretation of the qualitative and quantitative profile of phenolic compounds of unifloral honeys. In fact, when the analytical bias is statistically outside the interval of values typical for a known analyte concentration [90] a systematic error can affect the meaning of the conclusions obtained on the basis of such data. In the case of honey, the absence of suitable certificated reference materials (CRMs) and the frequent impossibility of comparing the results coming from the proposed procedure to the ones deriving from independent analytical methods, imply that the only feasible option for evaluating accuracy is represented by recovery tests. Unfortunately, even this approach may be problematic when increasing the number of analytes. On the other hand, precision is also an important data quality parameter. It is crucial to remember that precision varies with the level of analyte concentration [91] and depending on its measuring mode (e.g., within the same analytical session or in different analytical sessions), hence the evaluation of a single precision data is in general scarcely informative. With only few exceptions [58,59], the validation data reported in studies here considered are too often inconsistent or unreliable. In addition, no fitness-for-purpose assessment for precision and accuracy data has been evaluated, neither on the basis of the guidelines that have long been available in the literature [90,91].

## 3. Phenolic Profile as a Powerful Tool for Origin Classification and Evaluation of Health-Promoting Properties of Unifloral Honeys

### 3.1. Phenolic Compounds for the Classification of Botanical (or Geographical) Origin of Unifloral Honeys

Beyond being strictly associated to a number of health-promoting properties, the qualitative and quantitative profile of the phenolic compounds of unifloral honeys represents a powerful instrument for the verification of their origin. In the last years, the phenolic compounds of unifloral honeys have been characterized in order to identify components that—alone or, more easily, in combination with other compounds (phenolic or not)—could be descriptive of a specific floral origin. One of the most remarkable examples of specificity of a single phenolic acid as chemical marker of floral origin is still homogentisic acid for the strawberry tree honey [34,38,69]. Now more than 15 years since its discovery, this marker has never been found in any other unifloral honey, whereas many other candidate chemical markers have been later found also in honeys of other origins, thus losing any aspect of strict specificity towards a single unifloral honey. It is clear that almost all the scientific efforts made in this direction have been successful when devoted towards the quantification of one (or more) molecule rather than the presence/absence of a specific chemical marker. This is the case of methyl syringate, proposed as a marker of asphodel honey [56], but afterwards found in a number of different unifloral honeys [53,76,77,78,79], even if not in the same concentration levels. The research group of Tuberoso proposed additional chemical markers for the strawberry tree honey [69], Jerković and coworkers found useful chemical markers for unifloral honey by *Coffea* spp. [80], whereas, in a recent study on sage (*Sage officinalis* L.) honey from Croatia [67], four chemical species (*i.e.*, the flavonol kaempferol, present in quite high concentrations, boron and potassium among minerals, and turanose among sugars) were proposed as authentication markers for honeys of this botanical origin. Moreover, some compounds can be considerably useful to discriminate honeys which show similar properties and palinological features. This is the case of two typical honeys from New Zealand, like Manuka (*Leptospermum scoparium*) and Kanuka (*Kunzea erikoides*) honeys, which are indistinguishable by means of a melissopalynological analysis. In spite of the fact that Manuka and Kanuka honeys share most of the phenolic profiles, Stephens *et al.* [53] observed that 2-methoxybenzoic acid and trimethoxybenzoic acid are characteristic of Manuka honey while 4-methoxyphenylacetic acid is distinctive of the phenolic pattern of Kanuka honey.

Besides the characterization of honeys of a particular floral origin, the definition of phenolic profile has also been applied to investigate on honeys produced by subspecies of the common honeybee. For instance, some studies [73,92] have been devoted to the analytical characterization of honeys produced by the Sicilian black honeybee (*Apis mellifera* ssp. Sicula), with the identification and quantification of a number of phenolic acids and flavonoids. Furthermore, honeys produced by two different species of stingless honeybees have been investigated. More specifically, four phenolic acids, three flavonoids and the isomers of abscisic acid were identified and quantified in Jandaíra (*Melipona subnitida*) honey from Brazil [50], and the qualitative characterization of the O-glycosyl flavones of *Tetragonula carbonaria* honeys from Australia was performed in the already cited study by Truchado *et al.* [51].

Furthermore, attention has been focused on the use of the polyphenolic pattern to characterize honeys from particular geographical origins. In the study by Habib and coworkers [64] a significant difference in the content of phenolic compounds of honeys produced in non-arid and arid regions was found. The dissimilarity in the phenolic profile was explained on the basis of the different climate and sunlight exposure indicating the latter as the responsible for the higher content of polyphenols in honeys produced in arid regions. The phenolic profile of Serbian unifloral honeys was investigated by Kečkeš and coworkers [57], who suggested that quercetin and eriodictyol could be proposed as floral markers for local sunflower honeys. As a further example, phenolic profile of Sulla (*Hedysarum* spp.) honeys produced in Southern Italy resulted influenced by their geographical origin [93], whereas the concentrations of gallic, chlorogenic, caffeic, *p*-coumaric and ferulic acids showed the highest variation as a function of the production site of this honey.

As previously said, the definition of the qualitative and quantitative profile of phenolic compounds in unifloral honeys is surely suitable to give key information on their botanical and/or their geographical origin, but data obtained are reliable only if they are originated by an adequate number of samples. Since large data sets can be very difficult to be properly managed and correctly interpreted, a chemometric approach may be decisive to distinguish data from noise and to maximize analytical information [94]. Chemometrics is a powerful and interdisciplinary science finalized to extract and to maximize information from chemical systems by both descriptive and predictive viewpoints. Recently Gašić and coworkers [67] explored a number of classes of analytes (*i.e.*, polyphenolic profiles, the total phenolic contents, the compositions of minerals, sugars and sugar alcohols, and the radical scavenging activities), creating a dataset that was interpreted using PCA and targeted to the authentication of unifloral *Salvia officinalis* L. honey. Again, Petretto and coworkers [95] performed this methodological approach in classifying, by means of PCA, the botanical origin of fifty one Sardinian samples belonging to ten different kind of unifloral honeys according to the phenolic content, antioxidant power and physico-chemical properties. Also Kečkeš and coworkers [57] used PCA in order to rationalize the phenolic profile of forty four unifloral honey from Serbia, whereas—in a very recent contribution—Zhao *et al.* [81] used PCA and discriminant analysis (DA) to correctly classify more than 85% of the honey samples, according to their botanical origin. Kuś and van Ruth attempted the discrimination of Polish unifloral honeys using proton transfer reaction mass spectrometry (PTR-MS) and HPLC-DAD fingerprints combined with PCA and k-nearest neighbor classification [82]. Whereas models based on HPLC fingerprints may be useful as universal methods of classification, the model based exclusively on PTR-MS findings is only exploitable for quick targeted on-line screenings and for specific unifloral honeys. Chemometric models have also been used for the rationalization of data by analytical techniques other than HPLC. The already cited study performed by Jandric and coworkers [83] allowed to determine which technique (or combination of techniques) is able to provide the best classification and prediction abilities towards a group of authentic unifloral honeys from New Zealand. This result was accomplished using chemometric tools such as orthogonal partial least square discriminant analysis. In addition, the contribution of Lenhardt *et al.* [85] proposed a new method for unifloral honey characterization and classification based on fluorescence data treated with parallel factor analysis and partial least squares discriminant analysis.

Moreover, chemometric techniques allowed providing reliable information concerning the geographical origin of unifloral honeys also on the basis of their phenolic composition. This is the case of the study of Karabagias *et al.* [96], who differentiated, according to the geographical origin, thirty five samples of Greek thyme honey from four different sites. Differentiation was accomplished on the basis of the phenolic content and the conventional physicochemical parameters by means of multivariate analysis of the variance (MANOVA) and Linear Discriminant Analysis. Furthermore Pasquini *et al.* [97] used principal component analysis, discrimination methods, like linear and quadratic discriminant analysis, and classification and regression trees in order to accomplish geographic differentiation of fifty honey samples among its mineral contents, the total phenolic concentrations and the radical scavenging capacity. Classification and regression trees were found to be the model with the best predictive ability, specificity and sensitivity (66.67%, 80% and 67%, respectively).

Finally, the chemometric treatment (cluster analysis and PCA) of data from HPLC-ECD determination of the phenolic profile allowed Wang and coworkers to identify an acacia honey adulteration with rape honey [98]. In particular, chlorogenic acid and ellagic acid were hypothesized as possible markers of acacia and rape honeys, respectively.

### 3.2. Phenolic Compounds in the Health-Promoting Properties of Unifloral Honeys

The many health-promoting effects of honey have been well known for millennia. Beyond being the only form of sweetener available at that time, honey has been used by ancient cultures as medicine, but also as ointment. The traditional experience of our fathers is now supported by a solid scientific background that has been summarized in a number of authoritative and recent reviews [2,3,21,25,99,100]. Below we reported the principal health properties of honey directly attributed to the phenolic profile.

First, it has been ascertained that honey inhibits the growth of micro-organisms and fungi, and the botanical origin of honey is one of the most important factors influencing its antimicrobial activity. These properties have been attributed both to enzymes hydrogen peroxide-producers, like glucose oxidase and catalase, and non-peroxide substances, like the phenolic compounds. 

On the other hand, also the antioxidant action of the honey is well-known from ancient times. It has been associated to a number of different substances present in fresh honey, first of all enzymes and phenolic compounds, but also carotenoids derivatives, amino acids, proteins and—usually—low amounts of vitamins, all active against the so called “oxidative stress”. With this term the lack of equilibrium between the antioxidant protective activity in a given organism and the production of free radicals has been defined. There are several ways to measure *in vitro* the antioxidant activity of honey, and to compare it with the total amount of phenolic compounds [100]. Among the most effective methods there are the Ferric Reducing Antioxidant Power (FRAP) spectrophotometric assay, and the 2,2-DiPhenyl-1-PicrylHydrazyl (DPPH) radical scavenging method. In a recent contribution Moniruzzaman *et al.* exhaustively reviewed the most important analytical methods devoted to determine the antioxidant properties of honey [101].

There is an abundant literature concerning the evaluation of antimicrobial effects and antioxidant capabilities of unifloral honeys worldwide. These contributions describe the use of reference (or published) analytical methods to accomplish this task and they are often accompanied by the measure of some spectrophotometric parameters like the total polyphenolic and flavonoidic amounts and/or the colour. Less frequently, in these studies additional characterizations like a chromatographic phenolic profile, a melissopalynological analysis and a mineral composition of major and trace elements are present. In the last ten years, many unifloral honeys from New Zealand [23], Burkina Faso [26], Morocco [102], Italy [48,66,92,93,95,103,104,105,106,107], India [49,64], Brazil [50], United Arabian Emirates, Oman, Yemen, Pakistan, Australia [64], Japan [65], Croatia [67,79], Poland [76,78], Turkey [108,109], Portugal [110], Romania [111], Slovenia [112], Cuba [113,114,115,116], Greece [117] and Serbia [117] have been evaluated for their antimicrobial, antioxidants and radical scavenging properties evidencing their dependence on the presence of specific phenolic compounds like homogentisic acid, as well as on the botanical and geographical origin [93] and the climatic conditions [64]. Table 2 reports a selection of antioxidant and antiradical properties of the unifloral honeys described in last ten years.

In particular, Rosa *et al.* demonstrated that, among a group of seven honeys (*i.e.*, acacia, asphodel, *Citrus* spp, eucalyptus, heather, honeydew and strawberry tree) from Italy, the strawberry tree one showed the highest concentration in total phenols and the major activity in the DPPH and FRAP tests. The amount of homogentisic acid (*i.e.*, the chemical marker for strawberry tree honey) was more than 60% of the total phenols of such honey and this phenolic acid showed interesting antioxidant and antiradical activities as well as protective effect against thermal cholesterol degradation, comparable to those of a number of well-known antioxidants [105]. However researches performed by Tuberoso and coworkers [104], aimed to evaluate the antioxidant capacity and vasodilatory properties of three Mediterranean foods rich in phenolic compounds like the Cannonau red wine, myrtle berries liqueur and strawberry-tree honey, demonstrated that such honey—unlike to what shown by red wine and the liqueur—did not induce any vasodilation. This confirms the fact that the abundance of phenolic compounds in foods does not represent an assurance about their functional properties that have to be tested by suitable methods. Also unifloral honeys from Cuba have attracted a great interest in this last decade. For the first time the phytochemical composition of five important monofloral Cuban honeys and their possible relationships with their biological activities were thoroughly studied by Alvarez-Suarez *et al.* In particular, their antioxidant [113,114] and antimicrobial [114] properties were examined and discussed also in terms of correlation with colour, total amount of phenolic compounds and concentrations of flavonoid species, amino acids, proteins and carotenoids. The samples analysed possessed good antioxidant and antibacterial properties and meaningful concentrations of phenolic species, flavonoids and carotenoids. Later, the same research group successfully studied the protective effect of such unifloral honeys against lipid peroxidation in an *in vitro* model of rat liver homogenates [115] and the ability of the phenolic extracts of two unifloral honeys (*i.e.*, the Christmas vine, *Turbina corymbosa* L., and the Linen vine, *Gouania polygama*) to inhibit the oxidative damage induced by the 2,2’-azobis(2-methylpropionamidine) dihydrochloride in erythrocytes [116].

Recently, attention has been paid to alternative analytical approaches for assessing the antioxidant capacity of honey and relating it to the presence of polyphenolic species. In particular, Avila and coworkers proposed a novel, simple and fast electrochemical method devoted to estimate the antioxidant capacity in honey samples using flow injection analysis. The method is based on a selective electrooxidation of polyphenolic compounds using two different anodic potentials at two different pH values. The significance of the proposed protocol *vs.* the traditional spectrophotometric method was enhanced by a chemometric evaluation of data obtained. The procedure is inherently versatile, because it allows the evaluation of the antioxidant activity under predesigned oxidation conditions. Finally, an electrochemical antioxidant index is proposed for the evaluation of the antioxidant capabilities of honey [117]. This work demonstrates that electrochemistry is an emerging and valuable tool in the direct evaluation of antioxidant capacity of natural complex extracts from foods. Indeed, one year later, Buratti proposed a similar method to evaluate the antioxidant power of honeybee products (*i.e.*, honey, propolis and royal jelly) by an amperometric flow injection method [106]. Also this method is easy and rapid (one measure/minute). Finally, in 2013, Gorjanovića *et al.* published a contribution concerning the assessment of an electroanalytical method aimed to check the applicability of DC polarography in determination of hydrogen peroxide scavenging activity of honey [118]. The reported results, compared with selected antioxidant assays like DPPH, FRAP, TEAC and ORAC, demonstrated that the hydrogen peroxide scavenging activity is discriminant towards the floral origin of honeys. 

Still few contributions in assessing methods and interpreting results on antioxidant activity of unifloral honeys comes from the application of chemometric tools. In 2005, for the first time, Beretta and coworkers successfully attempted to standardize the antioxidant properties of honey by spectrophotometric and by fluorimetric data treated with multivariate techniques (correlation matrix calculation and PCA) [107]. In particular, Authors investigated the antioxidant power and the radical scavenging capacity of fourteen commercial honeys of different floral and geographic origin, using many of the known spectrophotometric tests like Folin-Ciocalteu assay for phenol content, FRAP assay, DPPH assay for antiradical activity, ORAC for the antilipoperoxidant activity, whereas color intensity was evaluated measuring absorbance at 450 nm. Multivariate techniques allowed to find significant correlations for all the antioxidant markers: antioxidant properties are strictly correlated to the phenolic content and honey colour intensity. Hence, it is evident that only with a chemometric approach for data obtained from the combination of different antioxidant assays it is possible to achieve reliable guidelines for the characterization of the antioxidant activity of honey. Finally, the study of Escuredo *et al.* [119] has applied for the first time the near infrared spectroscopy to the selection of antioxidant variables in honey. Calibration models for phenols, flavonoids, vitamin C, antioxidant capacity (DPPH), oxidation index and copper in unifloral honeys were obtained using the modified partial least squares regression method. Such models were optimised by cross-validation, and the best model was evaluated according to multiple correlation coefficient, standard error of cross-validation, ratio performance deviation and root mean standard error in the prediction set. Hence, near infrared spectroscopy can be considered as a rapid and reliable tool for the non-destructive evaluation of chemical and health-promoting parameters in honey. 

## 4. Conclusions

Honey is an important source of phenolic compounds, and the amount and the nature of phenolic acids and flavonoids is of great interest because they are responsible for a number of functional and nutraceutical properties typical of this natural food. Moreover, several and recent studies have also confirmed that the phenolic profile is strictly related to the botanical and—sometimes—the geographical origin of unifloral honeys, so it can be used as invaluable tool for classification and authentication. Taking this into account, many groups have intensified their efforts in order to develop new and reliable analytical protocols able to improve the qualitative and quantitative characterization of phenolic compounds in honey, but also to evaluate the bioactive properties of new unifloral honeys worldwide and to correlate them to a number of health-promoting features of this foodstuff. While recent studies have frequently been accompanied by at least a minimal validation, the application of chemometric instruments for the optimization of procedures of obtaining and managing the analytical data still appears insufficient, although the trend is in sharp increasing in the last years.

## Figures and Tables

**Table 1:** Features of recent chromatographic methods for the analysis of phenolic compounds in unifloral honey.

Technique	Stationary Phase (Length, mm × id, mm × Particle Size, µm)	Mobile Phase ^a^	Validation	Chemometrics	Floral/Geographical Origin of Honey	Analytes ^b^	Ref.
HPLC-ECD	Zorbax SB-C18 (150 × 4.6 × 5)	A: 4% CH_3_COOH (aq) B: MeOH	y		*Citrus* honey from China	Caffeic acid, *p*-coumaric acid, ferulic acid, hesperetin	[46]
HPLC-DAD	Wonda-Sil C18 (150 × 4.6 × 5)	MeOH 43% (*v*/*v*) and HCOOH (aq), pH 2.54 (57%, *v*/*v*)	y	Multivariate calibration	Milk vetch, wild chrysanthemum, jujube flower and acacia honeys from China	Gallic acid, chlorogenic acid, protocatechuic acid, caffeic acid, *p*-hydroxybenzoic acid syringic acid, *p*-coumaric acid, ferulic acid, rutin	[47]
HPLC-UV	Hypersil gold C18 (250 × 4.6 × 5)	A: KH_2_PO_4_ (aq), pH 2.92 B: MeOH	y		Chestnut, eucalyptus, citrus and sulla honeys from Italy	Gallic acid; gallocatechin; epicatechin; catechin; chlorogenic acid; caffeic acid;, benzoic acid; *p*-coumaric acid; ferulic acid; rutin; myricetin; quercetin	[48]
HPLC-UV	Nova-Pak C18, (150 × 3.9)	A: H_3_PO_4_ (aq) pH 2.5; B: MeCN	n	**	*Sesamum indicum* honey from Hooghly district, West Bengal	Rutin, quercetin, apigenin and myricetin, ferulic acid	[49]
HPLC-DAD	Shimpack CLC-ODS, (250 × 4.6 × 5)	A: 5% HCOOH (aq) B: MeOH	n		Jandaira honey from state of Paraiba, Brazil	Naringenin, quercetin, isorhamnetin, gallic acid, vanillic acid, 3,4-dihydroxybenzoic acid, cumaric acids, trans–trans abscisic acid, cis–trans abscisic acid	[50]
HPLC-DAD-MS^n^	LiChroCART RP-18 (250 × 4 × 5)	A: 1% HCOOH (aq) B: MeOH	n		Tetragonula carbonaria honey from Australia	*O*-glycosyl flavones	[51]
HPLC-UV	Spherisorb ODS-2	A: phosphate buffer (pH 2.92) B: MeOH	y		Chestnut, acacia, lime, eucalyptus, lavender, rapeseed, sunflower, rosemary, orange, lemon, fior di sulla, *Echium plantagineum*, heather, bell heather and ling heather honey from Germany, Denmark, Italy, Spain, France, Netherlands, UK and Portugal	Benzoic acid, salicylic acid, 3-hydroxybenzoic acid, 4-hydroxybenzoic acid, protocatechuic acid, gallic acid, syringic acid; vanillic acid; trans-cinnamic acid, o-; m- and p-coumaric acids, caffeic acid, ferulic acid, phenylacetic acid, l-β-phenyllactic acid, dl-*p*-hydroxy-phenyllactic acid	[52]
HPLC-MS/MS	Phenomenex Luna C-18 (150 × 2 × 3)	A: 0.2% HCOOH (aq) B: MeOH	n		Manuka and kanuka honeys from New Zealand	Gallic acid; syringic acid; 2-methoxybenzoic acid; phenyllactic acid; methyl syringate; abscisic acid; 4-methoxybenzoic acid; 4-methoxyphenyllactic acid (tentative identification); trimethoxybenzoic acid (tentative identification); structural isomer of syringic acid (tentative identification); 4-methoxyphenyllactic acid (tentative identification)	[53]
(1) HPLC-DAD (2) HPLC-MS/MS	Lichrocart Purosher Star RP-18e (250 × 4 × 5)	A: 0.2 M H_3_PO_4_ (aq) B: MeCN 2 Water:MeCN 60:40 (*v*/*v*)	n		Asphodel honey from Sardinia, Italy	Methyl syringate	[56]
UHPLC-HESI-MS^n^	Hypersil gold C18 (50 × 2.1 × 1.9)	A: 0.1% HCOOH (aq) B: 0.1% HCOOH (MeCN)	y	Analytical data are interpreted in terms of principal component analysis	Acacia, sunflower, linden, basil, buckwheat, oilseed rape and goldenrod honeys from Serbia	Gallic acid; protocatechuic acid; 3-*o*-caffeoylquinic acid; caffeic acid; quercetin-3-*o*-rutinoside; *p*-coumaric acid; luteolin; quercetin; 2-cis,4-trans-abscisic acid; apigenin; kaempferol; chrysin; pinocembrin; galangin	[57]
UHPLC–UV	Chromolith FastGradient RP-18e (2 × 50 × 2)	A: 0.1% HCOOH (aq) B: 0.1% HCOOH (MeOH)	y		Acacia, sulla, thistle and *citrus* honeys from Calabria, Italy	(±)-cis,trans-abscisic acid, apigenin, caffeic acid, chrysin, ferulic acid, hesperetin, pinobanksin, *p*-coumaric acid, quercetin, syringic acid, vanillic acid, galangin, kaempferol, luteolin, myricetin, pinocembrin	[58]
HPLC-DAD-TOF-MS	Discovery HS PEG (150 × 4.6 × 5)	A: 0.1% HCOOH (aq) B: MeCN	y		Lavender, orange blossom, rosemary, heather, eucalyptus, chestnut and thyme honeys. No declaration of geographical origin of honey was provided.	Baicalein, hesperetin, fisetin, naringenin, chrysin, myricetin, quercetin, kaempferol	[59]
HPLC-DAD	Betasil C18 (150 × 4.6 × 3)	A: 1% HCOOH (aq) B: MeOH	y		*Ziziphus Spina-Christi* honey from Yemen	Gallic acid; clorogenic acid; 4-hydroxybenzoic acid; 4-hydroxyphenyl acetic acid; caffeic acid; vanillic acid; syringic acid; *p*-coumaric acid; phenol; ferulic acid; sinapic acid; naringin; myricetin; quercetin; naringenin; cinnamic acid; kaempferol; apigenin; chrysin; galangin; thymol; carvacrol	[60]
UHPLC-MS	Acquity UHPLC BEH C18 (150 × 2.1 × 1.7)	A: 0.1% HCOOH (aq) B: Methanol	y		*Ziziphus Spina-Christi* honey from Yemen	Gallic acid, 4-hydroxybenzoic acid, 4-hydroxyphenyl acetic acid, caffeic acid, chlorogenic acid, vanillic acid, syringic acid, *p*-coumaric acid, ferulic acid, phenol, myricetin, naringin, cinnamic acid, quercetin, naringenin, kaempferol, luteolin, apigenin, galangin, chrysin	[61]
HPLC-UV	Waters Xterra RP 18 (150 × 4.6 × 5)	A: 1% CH_3_COOH (aq) B: MeCN	n		*Prosopis juliflora, Ziziphus Spina-Christi, Acacia tortilis* and *Leptospermum scoparium* honeys from UAE, Oman, Yemen and New Zealand	Gallic acid 4-hydroxy-3-methoxybenzoic acid; syringic acid *p*-coumaric acid; ferulic acid cinnamic acid; catechin, epicatechin, rutin	[64]
HPLC-DAD	Shiseido Capcell Pak C18 UG120, (250 × 4.6 × 5)	A: TFA 0.1% (aq) B: TFA 0.1% (MeOH)	n		Peppermint honey from USA.	*p*-coumaric acid and kaempferol	[65]
HPLC-UV	Whatman ODS-2 column (250 × 4*.*6 × 5)	A: 87/3/10 (*v*/*v*/*v*) water/TFA/MeCN B: 40/50/10 (*v*/*v*/*v*) water/TFA/MeCN	n		Acacia, chestnut, savory, sulla, ailanthus, thymus and orange honeys from Italy	Gallic acid; chlorogenic acid; *p*-coumaric acid; caffeic acid; myricetin; quercetin; genistein; kaempferol; apigenin; chrysin; galangin	[66]
UHPLC-DAD MS/MS	Syncronis C18 column (100 × 1 x 1.7)	A: 0.1% HCOOH (aq) B: MeCN	n	Analytical data are interpreted in terms of principal component analysis.	Sage honey from Croatia	Gallic acid; gallocatechin; protocatechuic acid; epigallocatechin; gentisic acid; *p*-hydroxybenzoic acid; chlorogenic acid; catechin; caffeic acid; gallocatechin gallate; epicatechin; *p*-coumaric acid; ferulic acid; rosmarinic acid; epigallocatechin gallate; cis,trans-abscisic acid; resveratrol; kaempferol; pinobanksin; quercetin; chrysin; pinostrobin; pinocembrin; hesperetin; galangin	[67]
HPLC-DAD	Phenomenex Gemini C18 110° (150 × 4.60 × 3)	A: 0.2 M H_3_PO_4_ (aq) B: MeCN	n		Thistle honey from Sardinia, Italy [68]	Phenyllactic acid [68]	[68]
HPLC-DAD HPLC-MS/MS	Phenomenex SynergiHydro-RP 80AC18 (150 × 4.60 × 4) Licrocart Purosher Star RP-18e	A: 0.2 M H_3_PO_4_ (aq) B: MeCN Water/MeCN 60:40 (*v*/*v*)	n		Strawberry tree honey from Sardinia, Italy	2-cis,4-trans-abscisic acid; 2-trans,4-trans-abscisic acid	[69]
HPLC-ESI-MS/MS	Atlantis C-18 (50 × 2.1 × 3)	A: 2 mM HCOOH (aq) B: MeOH	y		Buckwheat honey. No declaration of geographical origin of honey was provided.	Gallic acid, *p*-hydroxyphenylacetic, acid, *p*-hydroxybenzoic acid, chlorogenic acid, vanillic acid, caffeic acid, syringic acid, *p*-coumaric acid, ferulic acid, rutin, myricetin, naringin, naringenin, quercetin, apigenin	[70]
HPLC-DAD-ESI-MS/MS	C18 LiChroCART (250 × 4 × 5)	A: 1% CH_3_COOH (aq) B: MeOH	n		Canola, cherry blossom, eucalyptus, linden, lucerne, lavender, orange blossom, rapeseed, rhododendron, rosemary, sunflower, taraxacum and tilia honeys from Italy, Spain and Slovakia	Flavonoid glycosides	[71]
HPLC-CEADHPLC-ESI-MS	Nucleodur Sphinx RP (150 × 4.6 × 5)	^c^ ^d^	y		Acacia, buckwheat, maple, phacelia, pumpkin, raspberry, orange, cherry blossom, dandelion, melon, rhododendron, rosemary, citrus blossom, orange blossom, lavender, sage, thyme, pine tree and rape seed honeys from Italy, Austria, Croatia, Greece and Germany.	Quercetin, naringenin, hesperetin, luteolin, kaempferol, isorhamnetin, galangin	[72]
HPLC-DAD-MS^n^	Gemini C18 110 Å (150 × 2 × 5)	A: 0.1% HCOOH (aq) B: MeOH	n		Sulla, dill, lemon, orange, and medlar honeys from Sicily, Italy	4-methoxyphenyllactic acid; citric acid; phenylalanine; phenyllactic acid; dehydrovomifoliol; 3-hydroxy-1-(2-methoxyphenyl)penta-1,4-dione; *p-*hydroxybenzoic acid; riboflavin; kynurenic acid; methyl syringate; quercetin hexosyl rutinoside; quercetin rhamnosyl-hexosyl-rhamnoside; lumichrome; quercetin rutinoside; abscisic acid; pinobanksin methyl ether; kaempferol rutinoside; pinobanksin; pinocembrin; caffeic acid isoprenyl ester; pinobanksin acetate; pinobanksin butyrate.	[73]
UPLC-DAD-MS/MS	Nucleodur C18 Pyramid (100 × 2.1 × 1.8)	A: 0.1% HCOOH (aq) B: 0.05% HCOOH (MeCN)	n		Manuka honeys from New Zealand	Gallic acid, caffeic acid, phenyllactic acid, 4-methoxyphenyllactic acid, kojic acid, 4-hydroxybenzoic acid, syringic acid, 2-methoxybenzoic acid, phenylacetic acid, benzoic acid, methyl syringate, 2-trans,4-trans-abscisic acid, 2-cis,4-trans-abscisic acid, luteolin	[74]
MLC-UV	Spherisorb C18 column (250 × 4.6 × 10)	7.8%*v*/*v* EtOH and 5.0%*v*/*v* CH_3_COOH in water, [SDS] = 0.124 mol/L	y	Experimental design (CCD) was used to optimize the chromatographic separation	*Citrus* honey from Iran	Quercetin, hesperetin, chrysin	[75]
HPLC-DAD	Phenomenex Gemini C18 110° (150 × 4.60 × 3)	A: 0.2 M H_3_PO_4_ (aq) B: MeCN	n		Cornflower honey from Poland [76] Willow honey from Poland [77] *Black locust, rapeseed, lime, goldenrod, heather and buckwheat honeys from Poland* [78] Summer Savory honey from Poland [79]	Methyl syringate: phenyllactic acid [76] Gallic acid, benzoic acid, *p*-coumaric acid, 4-hydroxybenzoic acid, kynurenic acid, methylbenzaldehyde, methyl syringate, vanillic acid, (±)-2-trans,4-trans-abscisic acid [77] *p-hydroxybenzoic acid, methyl syringate, cis,trans-abscisic acid, gallic acid*[78] Methyl syringate [79]	[76,77,78,79]
HPLC-DAD	Phenomenex Kinetex C18 (150 × 4.60 × 5)	A: 0.2 M H_3_PO_4_ (aq) B: MeCN	y		Two samples of *Coffea* spp. honey of different geographical origin	Kojic acid	[80]
HPLC-DAD	RP-LiChrosorb Hibar C18 (250 × 2.4 × 5)	A: 5% HCOOH (aq) B: MeOH	n	Analytical data are interpreted in terms of principal component analysis.	Jujube, longan and chaste honeys from China	Catechin, chlorogenic acid, syringic acid, *p*-hydroxycinnamic acid, ferulic acid, rutin, trans-cinnamic acid, quercetin, kaempferol, apigenin, galangin, pinocembrin, pinobanksin	[81]
HPLC–ECD-DAD	Zorbax SB-C18 (250 × 4.6 × 5)	A: 1% CH_3_COOH (aq) B: MeOH	y	Honey classification has been accomplished by means of principal component analysis and discriminant analysis	Rapeseed, lime, heather, cornflower, buckwheat and black locust honeys from Poland	Gallic acid, protocatechuic acid, *p*-hydroxybenzoic acid, chlorogenic acid, vanillic acid, caffeic acid, syringic acid, *p*-coumaric acid, ferulic acid, sinapic acid, ellagic acid, rosmarinic acid, cinnamic acid	[82]

^a^ MeOH = methanol, MeCN = acetonitrile, TFA = trifluoroacetic acid; ^b^ only quantified analytes are indicated; ^c^ Phase A: MeOH/0.02 M phosphate buffer (20:80, *v*:*v*) pH 3.2, Phase B: MeOH/0.02 M phosphate buffer (80:20, *v*:*v*) pH 3.2; ^d^ Phase A: 0.5% CH_3_COOH in MeOH/water (20:80, *v*:*v*); Phase B: 0.5% CH_3_COOH in MeOH/water (80:20, *v*:*v*); y = yes; n = no; methods in italic provided only qualitative findings of analytes.

**Table 2:** Antioxidant and antiradical properties of unifloral honeys.

Floral and Geographical Origin of Unifloral Honey	Antioxidant and Antiradical Properties	Ref.
*Combretaceae* Honeys from Burkina Faso	DPPH, IC_50_ (mg/mL ± SD): between 10.40 ± 0.50 and 17.97 ± 1.44	[26]
	AEAC, (mg/100 g ± SD): between 16.34 ± 0.25 and 23.40 ± 0.74	
	QEAC, (mg/100 g ± SD): between 6.89 ± 2.02 and 11.31 ± 0.28	
*Acacia* Honey from Burkina Faso	DPPH, IC_50_ (mg/mL ± SD): 10.40 ± 0.50	
	AEAC, (mg/100 g ± SD): 23.40 ± 0.74	
	QEAC, (mg/100 g ± SD): 11.31 ± 0.28	
*Vitellaria* Honeys from Burkina Faso	DPPH, IC_50_ (mg/mL ± SD): between 1.37 ± 0.03 and 2.43 ± 0.08	
	AEAC, (mg/100 g ± SD): between 57.72 ± 0.00 and 65.86 ± 0.10	
	QEAC, (mg/100 g ± SD): between 31.01 ± 0.03 and 33.34 ± 0.21	
*Lannea* Honey from Burkina Faso	DPPH, IC_50_ (mg/mL ± SD): 23.53 ± 0.40	
	AEAC, (mg/100 g ± SD): 11.27 ± 0.02	
	QEAC, (mg/100 g ± SD): 5.35 ± 0.01	
*Amorpha fruticosa* honey from unknown geographical origin ^a^	DPPH, IC_50_ (mg/mL): 0.6 (data measured on pentane–diethyl ether (1:2, *v*/*v*) ultrasonic extracts from a 40% (*w*/*w*) aqueous solution of honey)	[27]
Chestnut honey from Italy	DPPH, I% (% ± SD): 75.37 ± 7.87	[48]
Eucalyptus honey from Italy	DPPH, I% (% ± SD): 73.04 ± 7.52	
Citrus honey from Italy	DPPH, I% (% ± SD): 55.06 ± 7.04	
Sulla honey from Italy	DPPH, I% (% ± SD): 66.60 ± 12.71	
*Sesamum indicum* honey from Hooghly district, West Bengal	DPPH, IC_50_ (mg/mL): 39.5 ± 0.4	[49]
	FRAP, (μmol Fe(II)/L): 2.75 × 10^6^	
Jandaíra honey from Brazil	DPPH, IC_50_ (mg/mL ± SD): between 10.6 ± 0.6 and 12.9 ± 0.3	[50]
	ABTS, IC_50_ (mg/mL ± SD): between 6.1 ± 0.0 and 9.7 ± 0.1	
*Prosopis juliflora* honeys from UAE ^b^	DPPH, I%: *ca.* 6	[64]
	ABTS, I%: *ca.* 74	
	FRAP, (μmol Fe(II)/100 g honey): *ca.* 800	
*Ziziphus Spina-Christi* honeys from UAE, Oman, Yemen and Pakistan^b^	DPPH, I%: between *ca.* 3 and *ca.*14	
	ABTS, I%: between *ca.* 75 and *ca.* 80	
	FRAP, (μmol Fe(II)/100 g honey): between *ca.* 600 and *ca.* 900	
*Acacia tortilis* honeys from UAE, Oman and Yemen ^b^	DPPH, I%: between *ca.*4 and *ca.*19	
	ABTS, I%: between *ca.* 72 and *ca.* 80	
	FRAP, (μmol Fe(II)/100 g honey): between *ca.* 500 and *ca.* 700	
*Leptospermum scoparium* honeys from New Zealand ^b^	DPPH, I%: *ca.* 11	
	ABTS, I%: *ca.* 79	
	FRAP, (μmol Fe(II)/100 g honey): *ca.* 600	
23 unifloral honeys from worldwide	DPPH, I%_,_ (% ± SD): between 4.7 ± 2.3 (Horse chestnut honey, Akita, Japan) and 51.9 ± 2.0 (Peppermint honey, USA)	[65]
Acacia honey from Italy	DPPH, IC_50_ (mg/mL ± SD): 21.56 ± 1.08	[66]
	FRAP, (mmol Fe(II)/Kg honey ± SD): 1.377 ± 0.068	
Chestnut honey from Italy	DPPH, IC_50_ (mg/mL ± SD): 13.76 ± 0.82	
	FRAP, (mmol Fe(II)/Kg honey ± SD): 2.056 ± 0.103	
Sulla honey from Italy	DPPH, IC_50_ (mg/mL ± SD): 54.74 ± 3.28	
	FRAP, (mmol Fe(II)/Kg honey ± SD): 1.299 ± 0.080	
Ailanthus honey from Italy	DPPH, IC_50_ (mg/mL ± SD): 64.09 ± 2.56	
	FRAP, (mmol Fe(II)/Kg honey ± SD): 1.268 ± 0.063	
Thymus honey from Italy	DPPH, IC_50_ (mg/mL ± SD): 31*.*4 ± 1*.*57	
	FRAP, (mmol Fe(II)/Kg honey ± SD): 1.834 ± 0.092	
Orange honey from Italy	DPPH, IC_50_ (mg/mL ± SD): 25.87 ± 1.29	
	FRAP, (mmol Fe(II)/Kg honey ± SD): 1.265 ± 0.063	
Savory honey from Italy	DPPH, IC_50_ (mg/mL ± SD): 10.85 ± 0.43	
	FRAP, (mmol Fe(II)/Kg honey ± SD): 3.702 ± 0.185	
Cornflower honey from Poland	DPPH, (mmol TEAC/kg ± SD): 0.5 ± 0.2	[76]
	FRAP, (mmol Fe(II)/Kg honey ± SD): 1.5 ± 0.7	
Willow honey from Poland	DPPH, (mmol TEAC/kg ± SD): 2.1 ± 0.3	[77]
	FRAP, (mmol Fe(II)/Kg honey ± SD): 0.5 ± 0.1	
Black locust honey from Poland	DPPH, (mmol TEAC/kg ± SD): 0.3 ± 0.0	[78]
	FRAP, (mmol Fe(II)/Kg honey ± SD): 0.6 ± 0.1	
Rapeseed honey from Poland	DPPH, (mmol TEAC/kg ± SD): 0.4 ± 0.1	
	FRAP, (mmol Fe(II)/Kg honey ± SD): 1.3 ± 0.3	
Lime honey from Poland	DPPH, (mmol TEAC/kg ± SD): 0.4 ± 0.1	
	FRAP, (mmol Fe(II)/Kg honey ± SD): 1.4 ± 0.4	
Goldenrod honey from Poland	DPPH, (mmol TEAC/kg ± SD): 0.2 ± 0.1	
	FRAP, (mmol Fe(II)/Kg honey ± SD): 1.0 ± 0.1	
Heather honey from Poland	DPPH, (mmol TEAC/kg ± SD): 0.6 ± 0.1	
	FRAP, (mmol Fe(II)/Kg honey ± SD): 2.1 ± 0.5	
Buckwheat honey from Poland	DPPH, (mmol TEAC/kg ± SD): 1.2 ± 0.2	
	FRAP, (mmol Fe(II)/Kg honey ± SD): 5.7 ± 0.9	
Summer Savory honey from Poland	DPPH, (mmol TEAC/kg ± SD): 1.7 ± 0.2	[79]
	FRAP, (mmol Fe(II)/Kg honey ± SD): 4.3 ± 0.6	
Sulla honeys from Southern Italy	DPPH, (I% ± SD): between 47.06 ± 8.60 and 88.25 ± 9.85	[93]
	FRAP, (μmol Fe(II)/100 g honey): between 98.26 ± 28.61 and 786.53 ± 91.28	
Strawberry tree honey from Sardinia ^b^	DPPH, (mg TE/100 g honey): *ca.* 51	[95]
	FRAP, (mg TE/100 g honey): *ca.* 89	
Asphodel honey from Sardinia ^b^	DPPH, (mg TE/100 g honey): *ca.* 4.5	
	FRAP, (mg TE/100 g honey): *ca.* 4	
Cardoon honey from Sardinia ^b^	DPPH, (mg TE/100 g honey): *ca.* 6	
	FRAP, (mg TE/100 g honey): *ca.* 6	
Eucalyptus honey from Sardinia ^b^	DPPH, (mg TE/100 g honey): *ca.* 8	
	FRAP, (mg TE/100 g honey): *ca.* 7	
Thymus honey from Sardinia ^b^	DPPH, (mg TE/100 g honey): *ca.* 4	
	FRAP, (mg TE/100 g honey): *ca.* 3	
Chestnut honey from Sardinia ^b^	DPPH, (mg TE/100 g honey): *ca.* 6.5	
	FRAP, (mg TE/100 g honey): *ca.* 8	
Cistus honey from Sardinia ^b^	DPPH, (mg TE/100 g honey): *ca.* 5.5	
	FRAP, (mg TE/100 g honey): *ca.* 7	
Lavender honey from Sardinia ^b^	DPPH, (mg TE/100 g honey): *ca.* 5	
	FRAP, (mg TE/100 g honey): *ca.* 4	
Rosemary honey from Sardinia ^b^	DPPH, (mg TE/100 g honey): *ca.* 7	
	FRAP, (mg TE/100 g honey): *ca.* 5.5	
Acacia honey from Morocco	DPPH, (mmol TE/Kg honey ± SD): 0.52 ± 0.01	[102]
	FRAP, (mmol Fe(II)/Kg honey ± SD): 2.15 ± 0.21	
Eucalyptus honey from Morocco	DPPH, (mmol TE/Kg honey ± SD): 0.68 ± 0.01	
	FRAP, (mmol Fe(II)/Kg honey ± SD): 2.99 ± 0.09	
Strawberry tree honey from Italy	DPPH, (mmol TE/Kg honey ± SD): 4.5 ± 1.1	[104]
	ABTS, (mmol TE/Kg honey ± SD): 5.9 ± 1.5	
	FRAP, (mmol Fe(II)/kg honey ± SD): 12.0 ± 2.2	
Strawberry tree honey from Italy ^b^	DPPH, (mmol TE/Kg honey): *ca.* 4.7	[105]
	FRAP, (mmol Fe(II)/kg honey): *ca.* 11.7	
Heather honey from Italy ^b^	DPPH, (mmol TE/Kg honey): *ca.* 1.45	
	FRAP, (mmol Fe(II)/kg honey): *ca.* 4.9	
Eucalyptus honey from Italy ^b^	DPPH, (mmol TE/Kg honey ): *ca.* 0.45	
	FRAP, (mmol Fe(II)/kg honey): *ca.* 3.0	
Asphodel honey from Italy ^b^	DPPH, (mmol TE/Kg honey): *ca.* 0.45	
	FRAP, (mmol Fe(II)/kg honey): *ca.* 4.3	
Citrus honey from Italy ^b^	DPPH, (mmol TE/Kg honey): *ca.* 0.3	
	FRAP, (mmol Fe(II)/kg honey): *ca.* 1.65	
Acacia honey from Italy ^b^	DPPH, (mmol TE/Kg honey): *ca.* 0.1	
	FRAP, (mmol Fe(II)/kg honey): *ca.* 0.55	
Citrus honey from Italy	DPPH, IC_50_ (mg/mL ± SD): between 5.0 ± 0.3 and 15.1 ± 0.4	[106]
Rhododendron honey from Italy	DPPH, IC_50_ (mg/mL ± SD): between 5.7 ± 0.3 and 15.5 ± 0.8	
Acacia honey from Italy	DPPH, IC_50_ (mg/mL ± SD): between 8± 1 and 12.0 ± 0.6	
Strawberry tree honey from Italy	FRAP, (μmol Fe(II)/Kg honey ± SD): 1501.4 ± 60.2	[107]
	DPPH, IC_50_ (mmol TE/Kg honey ± SD): 1.63 ± 0.17	
	ORAC, (mmol TE/Kg honey ± SD): 21.07 ± 0.34	
Buckwheat honey from Italy	FRAP, (μmol Fe(II)/Kg honey ± SD): 800.7 ± 23.8	
	DPPH, IC_50_ (mmol TE/Kg honey ± SD): 4.00 ± 0.44	
	ORAC, (mmol TE/Kg honey ± SD): 11.60 ± 0.027	
Chestnut honey from Italy	FRAP, (μmol Fe(II)/Kg honey ± SD): 388.6 ± 8.2	
	DPPH, IC_50_ (mmol TE/Kg honey ± SD): 7.93 ± 0.04	
	ORAC, (mmol TE/Kg honey ± SD): 8.90 ± 0.45	
Sulla honey from Italy	FRAP, (μmol Fe(II)/Kg honey ± SD): 155.2 ± 6.6	
	DPPH, IC_50_ (mmol TE/Kg honey ± SD): 16.90 ± 0.11	
	ORAC, (mmol TE/Kg honey ± SD): 5.66 ± 0.13	
Clover honey from Italy	FRAP, (μmol Fe(II)/Kg honey ± SD): 72.8±3.0	
	DPPH, IC_50_ (mmol TE/Kg honey ± SD): 25.00 ± 0.01	
	ORAC, (mmol TE/Kg honey ± SD): 2.15 ± 0.02	
Dandelion honeys from Italy	FRAP, (μmol Fe(II)/Kg honey ± SD): from 212.2±2.2 to 224.4±6.0	
	DPPH, IC_50_ (mmol TE/Kg honey ± SD): from 24.39 ± 0.07 to 47.62 ± 0.39	
	ORAC, (mmol TE/Kg honey ± SD): from 2.00 ± 0.02 to 7.59 ± 0.60	
Chicory honey from Italy	FRAP, (μmol Fe(II)/Kg honey ± SD): 209.5±2.8	
	DPPH, IC_50_ (mmol TE/Kg honey ± SD): 5.81 ± 0.04	
	ORAC, (mmol TE/Kg honey ± SD): 6.72 ± 0.33	
Acacia honey from Italy	FRAP, (μmol Fe(II)/Kg honey ± SD): 79.5±3.7	
	DPPH, IC_50_ (mmol TE/Kg honey ± SD): 45.45 ± 0.04	
	ORAC, (mmol TE/Kg honey ± SD): 2.12 ± 0.01	
Rosemary honey from Portugal	DPPH, IC_50_ (mg/mL ± SD): 168.94 ± 19.20	[110]
Viper’s bugloss honey from Portugal	DPPH, IC_50_ (mg/mL ± SD): 130.49 ± 1.38	
Heather honey from Portugal	DPPH, IC_50_ (mg/mL ± SD): 106.67 ± 2.48	
Acacia honey from Romania	DPPH, I%: between 35.80 and 45.27	[111]
Sunflower honey from Romania	DPPH, I%: between 36.60 and 40.91	
Lime honey from Romania	DPPH, I%: between 40.65 and 49.19	
Acacia honey from Slovenia	DPPH, IC_50_ (mg/mL): between 33.9 and 63.9	[112]
	FRAP, (μmol Fe(II)/100 g honey): between 56.8 and 86.0	
Lime honey from Slovenia	DPPH, IC_50_ (mg/mL): between 20.6 and 36.1	
	FRAP, (μmol Fe(II)/100 g honey): between 94.6 and 155.1	
Chestnut honey from Slovenia	DPPH, IC_50_ (mg/mL): between 7.8 and 14.0	
	FRAP, (μmol Fe(II)/100 g honey): between 238.3 and 469.5	
Fir honey from Slovenia	DPPH, IC_50_ (mg/mL): between 6.4 and 11.7	
	FRAP, (μmol Fe(II)/100 g honey): between 320.8 and 582.2	
Spruce honey from Slovenia	DPPH, IC_50_ (mg/mL): between 5.4 and 9.7	
	FRAP, (μmol Fe(II)/100 g honey): between 277.5 and 495.4	
Linen vine honey from Cuba	ORAC, (μmol of TE/g honey ± SD): 12.89 ± 0.28	[113]
	ABTS, (μmol of TE/g honey ± SD): 2.94 ± 0.23	
Morning glory honey from Cuba	ORAC, (μmol of TE/g honey ± SD): 9.26 ± 0.46	
	ABTS, (μmol of TE/g honey ± SD): 2.01 ± 0.21	
Singing bean honey from Cuba	ORAC, (μmol of TE/g honey ± SD): 8.12 ± 0.23	
	ABTS, (μmol of TE/g honey ± SD): 1.95 ± 0.14	
Black mangrove honey from Cuba	ORAC, (μmol of TE/g honey ± SD): 7.45 ± 0.37	
	ABTS, (μmol of TE/g honey ± SD): 1.22 ± 0.24	
Christmas vine honey from Cuba	ORAC, (μmol of TE/g honey ± SD): 4.59 ± 0.51	
	ABTS, (μmol of TE/g honey ± SD): 1.03 ± 0.28	
Linen vine honey from Cuba	AEAC, (mg/100 g honey ± SD): 29.54 ± 1.62	[114]
	QEAC, (mg/100 g honey ± SD): 13.73 ± 1.32	
	DPPH, IC_50_ (mg/mL ± SD): 7.23 ± 1.17	
	TBARS, IC_50_ (mg/mL ± SD): 3.76 ± 0.42	
	Lipid hydroperoxides, (mmol ± SD): 32 ± 2.35	
Morning glory honey from Cuba	AEAC, (mg/100 g honey ± SD): 16.14 ± 1.21	
	QEAC, (mg/100 g honey ± SD): 7.34 ± 1.40	
	DPPH, IC_50_ (mg/mL ± SD): 15.88 ± 1.57	
	TBARS, IC_50_ (mg/mL ± SD): 6.47 ± 0.72	
	Lipid hydroperoxides, (mmol ± SD): 39 ± 3.26	
Singing bean honey from Cuba	AEAC, (mg/100 g honey ± SD): 19.7 ± 0.86	
	QEAC, (mg/100 g honey ± SD): 8.95 ± 0.49	
	DPPH, IC_50_ (mg/mL ± SD): 12.44 ± 1.56	
	TBARS, IC_50_ (mg/mL ± SD): 7.17 ± 0.52	
	Lipid hydroperoxides, (mmol ± SD): 46 ± 3.82	
Black mangrove honey from Cuba	AEAC, (mg/100 g honey ± SD): 14.65 ± 1.03	
	QEAC, (mg/100 g honey ± SD): 6.65 ± 0.52	
	DPPH, IC_50_ (mg/mL ± SD): 15.53 ± 1.11	
	TBARS, IC_50_ (mg/mL ± SD): 7.28 ± 1.03	
	Lipid hydroperoxides, (mmol ± SD): 43 ± 2.48	
Christmas vine honey from Cuba	AEAC, (mg/100 g honey ± SD): 10.85 ± 1.47	
	QEAC, (mg/100 g honey ± SD): 4.93 ± 0.74	
	DPPH, IC_50_ (mg/mL ± SD): 18.53 ± 1.92	
	TBARS, IC_50_ (mg/mL ± SD): 9.94 ± 1.31	
	Lipid hydroperoxides, (mmol ± SD): 51 ± 3.26	
Pine honey from Greece	FRAP, (mmol TE/Kg honey ± SD): 4.05 ± 0.03	[117]
	ORAC, (mmol TE/Kg honey ± SD): 11.6 ± 0.2	
	TEAC, (mmol TE/Kg honey ± SD): 5.06 ± 0.02	
	DPPH, IC_50_ (mmol TE/Kg honey ± SD): 1.18 ± 0.03	
Dead nettle honey from Serbia	FRAP, (mmol TE/Kg honey ± SD): 2.03 ± 0.03	
	ORAC, (mmol TE/Kg honey ± SD): 10.2 ± 0.3	
	TEAC, (mmol TE/Kg honey ± SD): 3.70 ± 0.04	
	DPPH, IC_50_ (mmol TE/Kg honey ± SD): 0.49 ± 0.01	
Linden honey from Serbia	FRAP, (mmol TE/Kg honey ± SD): 0.61 ± 0.02	
	ORAC, (mmol TE/Kg honey ± SD): 9.5 ± 0.1	
	TEAC, (mmol TE/Kg honey ± SD): 2.04 ± 0.06	
	DPPH, IC_50_ (mmol TE/Kg honey ± SD): 0.25 ± 0.01	
Acacia honey from Serbia	FRAP, (mmol TE/Kg honey ± SD): from 0.20 ± 0.00 to 0.26 ± 0.01	
	ORAC, (mmol TE/Kg honey ± SD): from 5.9 ± 0.1 to 6.5 ± 0.3	
	TEAC, (mmol TE/Kg honey ± SD): from 1.00 ± 0.02 to 1.02 ± 0.03	
	DPPH, IC_50_ (mmol TE/Kg honey ± SD): 0.00 ± 0.00	

Acronyms meaning: FRAP: ferric reducing antioxidant power; DPPH: 2,2-diphenyl-1-picrylhydrazyl radical; TEAC: Trolox equivalent antioxidant capacity; ORAC: oxygen radical absorbance capacity; AEAC, ascorbic acid equivalent antioxidant content; QEAC, quercetin equivalent antioxidant content; TBARS, thiobarbituric reactive substances; TEAC, Trolox equivalent antioxidant capacity; IC_50_, 50% inhibitory concentration; TE: Trolox equivalents. ^a^ IC_50_ for the honey samples was undeterminable (at the maximum concentration of honey in water (45 g/L), I% it was measured only a 25% DPPH inhibition); ^b^ Values roughly inferred by figures reported by authors.

## Abbreviations

The following abbreviations are used in this manuscript:

HMF	5-Hydroxymethyl-2-furaldehyde
HPLC	High Pressure Liquid Chromatography
SPE	Solid Phase Extraction
p-HBA	*para*-HydroxyBenzoic Acid
LLE	Liquid-Liquid Extraction
TLC	Thin Layer Chromatography
UHPLC	Ultra High Pressure Liquid Chromatography
HESI	Heated ElectroSpray Interface
MS	Mass Spectrometry
DLLME	Dispersive Liquid-Liquid MicroExtraction
UV	UltraViolet spectrophotometry detector
DAD	Diode Array Detector
TOF	Time Of Flight detector
MWCNT	MultiWalled Carbon NanoTubes
ECD	ElectroChemical Detector
RSD	Relative Standard Deviation
ASE	Accelerated Solvent Extraction
RP	Reverse Phase
MeOH	Methanol
MeCN	Acetonitrile
TFA	TriFluoroAcetic acid
NIR	Near InfraRed spectroscopy
FT-IR	Fourier Transform InfraRed spectroscopy
ISO	International Organization for Standardization
IEC	International Electrotechnical Commission
EC	European Community
FAO	Food and Agriculture Organization
LOD	Limit Of Detection
LOQ	limit Of Quantification
CRM	Certificated Reference Material
PCA	Principal Components Analysis
DA	Discriminant Analysis
PTR	Proton Transfer Reaction
MANOVA	Multivariate Analysis Of the Variance
FRAP	Ferric (ion) Reducing Antioxidant Power
TPTZ	2,4,6-tripyridyl-s-triazine
DPPH	2,2-diphenyl-1-picrylhydrazyl radical
TEAC	Trolox equivalent antioxidant capacity
ORAC	Oxygen Radical Absorbance Capacity
QEAC	Quercetin Equivalent Antioxidant Content
AEAC	Ascorbic acid Equivalent Antioxidant Content
TBARS	ThioBArbituric Reactive Substances
IC_50_	50% Inhibitory Concentration
